# EFFECTS OF ENVIRONMENTAL FACTORS ON THE INCIDENCE OF DEPRESSION IN OLDER ADULTS IN TAIWAN: A MULTI-LEVEL ANALYSIS

**DOI:** 10.1093/geroni/igz038.1006

**Published:** 2019-11-08

**Authors:** Chia Chun Li, Der-Chiang Li, Susan C Hu

**Affiliations:** National Cheng Kung University, Tainan City, Taiwan

## Abstract

Depression is a common mental disorder worldwide, and also a leading cause of disability and global burden of disease. The risk factors of depression are multi-facets; however, past studies have focused more on individual factors than ecological factors. This study aims to use a health map framework with multi-level analysis to analyze the effects of environmental factors on the incidence of depression of older adults in Taiwan. We connected 3 national datasets, including the National Health Interview Survey (NHIS), Age-friendly Dataset at city-level, and the National Health Insurance Research Databases (NHRID), to examine the relationship between environmental factors and the incidence of depression in Taiwan. The dependent variable was the new cases of depression in older adults during 2010-2016. The study variables were environment factors including 5 build environments and 8 social environments. A total of 6494 valid participants aged 50 and over were recruited from the 2009 NHIS and were followed up to 2016. There were 292 (4.5%) new incident cases of depression during 2010-2016. Results of multi-level analysis showed that only one built environment (no. of heritage sites) was associated with the incidence of depression. No social factors was found significantly in the study. It seems that factors of individual-level appear more important than that of ecological-level as the determinants of depression. Results of this study could provide an exploratory information for future research regarding the relationships between depression and environments.

